# Role of mitochondrial alterations in human cancer progression and cancer immunity

**DOI:** 10.1186/s12929-023-00956-w

**Published:** 2023-07-31

**Authors:** Sheng-Fan Wang, Ling-Ming Tseng, Hsin-Chen Lee

**Affiliations:** 1 Department of Pharmacy, Taipei Veterans General Hospital, No. 201, Sec. 2, Shipai Rd., Beitou Dist., Taipei, 112 Taiwan; 2 School of Pharmacy, Taipei Medical University, No. 250, Wuxing St., Xinyi Dist., Taipei, 110 Taiwan; 3 Department and Institute of Pharmacology, College of Medicine, National Yang Ming Chiao Tung University, No. 155, Sec. 2, Li-Nong St., Beitou Dist., Taipei, 112 Taiwan; 4 Division of General Surgery, Department of Surgery, Comprehensive Breast Health Center, Taipei Veterans General Hospital, No. 201, Sec. 2, Shipai Rd., Beitou Dist., Taipei, 112 Taiwan; 5 Department of Surgery, College of Medicine, National Yang Ming Chiao Tung University, No. 155, Sec. 2, Li-Nong St., Beitou Dist., Taipei, 112 Taiwan; 6 Department of Pharmacy, College of Pharmaceutical Sciences, National Yang Ming Chiao Tung University, No. 155, Sec. 2, Li-Nong St., Beitou Dist., Taipei, 112 Taiwan

**Keywords:** Mitochondria, Cancer progression, Retrograde signaling, Cancer immunity

## Abstract

Dysregulating cellular metabolism is one of the emerging cancer hallmarks. Mitochondria are essential organelles responsible for numerous physiologic processes, such as energy production, cellular metabolism, apoptosis, and calcium and redox homeostasis. Although the “Warburg effect,” in which cancer cells prefer aerobic glycolysis even under normal oxygen circumstances, was proposed a century ago, how mitochondrial dysfunction contributes to cancer progression is still unclear. This review discusses recent progress in the alterations of mitochondrial DNA (mtDNA) and mitochondrial dynamics in cancer malignant progression. Moreover, we integrate the possible regulatory mechanism of mitochondrial dysfunction–mediated mitochondrial retrograde signaling pathways, including mitochondrion-derived molecules (reactive oxygen species, calcium, oncometabolites, and mtDNA) and mitochondrial stress response pathways (mitochondrial unfolded protein response and integrated stress response) in cancer progression and provide the possible therapeutic targets. Furthermore, we discuss recent findings on the role of mitochondria in the immune regulatory function of immune cells and reveal the impact of the tumor microenvironment and metabolism remodeling on cancer immunity. Targeting the mitochondria and metabolism might improve cancer immunotherapy. These findings suggest that targeting mitochondrial retrograde signaling in cancer malignancy and modulating metabolism and mitochondria in cancer immunity might be promising treatment strategies for cancer patients and provide precise and personalized medicine against cancer.

## Background

Mitochondria are intracellular organelles with double membranes and their own genome in eukaryotic cells [1]. In mammalian cells, the fundamental function of mitochondria is to supply energy by adenosine triphosphate (ATP) production through the tricarboxylic acid (TCA) cycle and oxidative phosphorylation (OXPHOS) [2]. Mitochondria are also involved in several intermediate metabolism pathways, including glucose metabolism, fatty acid β-oxidation, and amino acid metabolism [3]. Moreover, mitochondria play critical roles in many physiological processes, such as apoptosis and redox or calcium homeostasis [4]. In addition, mitochondria-derived reactive oxygen species (ROS), which are byproducts of OXPHOS, were proposed to contribute to several diseases, such as aging, neurodegenerative disease, diabetes, and cancer [5].

Mitochondria harbor multiple copies of mitochondrial DNA (mtDNA) in the matrix near the inner membrane [6]. Human mtDNA is a double-stranded, circular DNA molecule of 16.6 kb and contains the genes encoding 2 rRNAs, 22 tRNAs, and 13 polypeptides responsible for the subunits of the electron transport chain (ETC) and ATP synthase in the OXPHOS system [6]. Because most mitochondrial proteins are encoded by nuclear genes, coordinative regulation of gene expression between mitochondrial and nuclear genomes and the import of proteins into mitochondria are crucial for mitochondrial biogenesis and maintenance [7].

Deregulation of cellular energetics has been recognized as a cancer characteristic [8]. Cancer cells preferentially utilize glycolysis over mitochondrial OXPHOS even in aerobic circumstances, also called the Warburg effect [9]. Further studies proposed that rather than impaired mitochondria, mitochondrial respiration in cancer cells might be insufficient [10, 11]. Decreased cellular respiration might not be essential for cancer cell proliferation, and the tumor microenvironment might be another critical factor for cancer progression [12]. The regulatory mechanisms leading to decreased cellular respiration in cancer cells are complicated and may depend on tumor type.

### Mitochondrial function regulates cancer metabolism remodeling and tumor microenvironment

Mitochondria play diverse roles in cancer metabolism. Mitochondria are essential not only in the process of energetic ATP synthesis via OXPHOS but also in lipid metabolism, amino acid metabolism, the TCA cycle, and nucleic acid metabolism [13]. Mutations in mtDNA and in the nuclear genes for the TCA cycle are commonly observed in cancer cells and are involved in cancer metabolism remodeling [13, 14]. Additionally, mutations in oncogenes and tumor suppressor genes, such as phosphatidylinositol 3-phosphate kinase (PI3K)-AKT-mammalian target of rapamycin (mTOR), MYC, RAS, and hypoxia-inducible factor 1 (HIF-1), might contribute to cancer metabolism remodeling via altered mitochondrial metabolic pathways such as OXPHOS and fatty acid, glutamine, and one-carbon metabolisms [15]. Metabolism reprogramming was thus proposed as one of the cancer hallmarks [8].

The Warburg effect leads cancer cells to preferring the generation of 2 ATP via aerobic glycolysis instead of OXPHOS. To compensate for ATP production, glycolysis is upregulated by increased glucose transporters (including glucose transporter 1) and glycolytic enzymes such as hexokinase-2 and lactate dehydrogenase-A. In addition, increased glycolytic intermediates might contribute to cancer cell proliferation via various biosynthetic pathways, such as the pentose phosphate pathway and one-carbon metabolism [16].

The TCA cycle, which occurs in the mitochondrial matrix, utilizes different nutrients (including glucose, fatty acids, and glutamine) to generate ATP or to convert macromolecules for biosynthesis. The hypoxic tumor microenvironment might contribute to fueling the TCA cycle with glutamine instead of pyruvate [17]. Mutations in TCA cycle enzymes are frequently observed in cancer and might be associated with cancer progression through cancer metabolism reprogramming and oncometabolite production [15]. Some cancer cells are glutamine addicted and prefer fuel for the TCA cycle. Glutamine can be converted to glutamate by glutaminase (GLS) and metabolized to α-ketoglutarate (α-KG) by glutamate dehydrogenase (GDH). Glutamate can also be metabolized to alanine/aspartate and α-KG by aspartate aminotransferase 2 (GOT2) and mitochondrial glutamate-pyruvate transaminase 2 (GPT2). Several lines of evidence have shown that alterations in GLS, GDH, GOT2, and GPT2 might be necessary for glutamine metabolism remodeling in cancer [15].

Fatty acid oxidation is another crucial metabolic process in the mitochondrial matrix [18]. Carnitine palmitoyltransferase (CPT) 1/2 is responsible for fatty acid transport into mitochondria. Fatty acid synthesis-related acetyl carboxylase (ACC) 1 or 2 can produce malonyl-CoA and inhibit CPT1. Some lines of evidence have shown that ACC1/2, CPT1A, and MYC alterations might be critical to lipid metabolism remodeling in cancer [15].

One-carbon metabolism (including the synthesis of thymidylate, methionine, serine/glycine, and purine) connects cytosolic and mitochondrial metabolism and is responsible for the methionine and folate cycle [19]. Several alterations in the mitochondrial folate cycle (such as serine hydroxymethyltransferase 2, methylenetetrahydrofolate dehydrogenase 2, and monofunctional tetrahydrofolate synthase 1L) and mitochondrial serine/glycine metabolism (glycine decarboxylase) might contribute to one-carbon metabolism reprogramming in cancer metabolism [15].

The tumor microenvironment is varied with several factors, including nutrients, oxygen content, acidic circumstances, and oxidative stress, among cancer cells, stromal cells, and immune cells [20]. Proliferative cancer cells exhaust all nutrients and oxygen very quickly, resulting in nutrient deprivation and hypoxia. In addition, lactate, a glycolytic product of cancer cells, contributes to acidic conditions. Although the Warburg effect proposes a preference for aerobic glycolysis, most cancer cells have intact mitochondria [21], and an energetic shift between glycolysis and OXPHOS can occur in cancer cells [22, 23]. Elevated ROS in the tumor microenvironment could originate from mitochondrial dysfunction, oncogenes, or dysregulated enzymes such as nicotinamide adenine dinucleotide phosphate oxidases, cycloxygenases, lipoxygenases, and thymidine phosphorylase [24]. The tumor microenvironment in progressed cancer might be detrimental to mitochondria due to limited oxygen levels, restricted nutrients, and high oxidative stress [25]. Therefore, mitochondrial alterations might contribute to cancer progression.

### Mitochondrial alterations might contribute to cancer progression

The alterations of mitochondria-related genes (including nuclear gene- and mtDNA-encoded), mitochondrial dynamics, mitochondrial content, and mitochondrial activity were usually observed in cancer cells, which have been intensively reviewed [13, 26–28]. The recent findings on the mtDNA alterations and mitochondrial dynamics in cancer progression are discussed.

### MtDNA alterations might lead to mitochondrial dysfunction and be the driving force behind carcinogenesis

MtDNA is a vulnerable genome due to the absence of efficient DNA repair systems and its location near the generation sites of ROS as byproducts of OXPHOS [29]. Accumulated mtDNA mutation-mediated mitochondrial dysfunction might enhance oxidative stress, forming a vicious cycle of mitochondrial dysfunction [30]. Several types of mtDNA alterations, such as point mutations, insertions, large-scale deletions, and copy number changes, have been detected in cancers [31–33]. Recently, comprehensive mitochondrial genome alterations in cancers were analyzed with the next-generation sequencing technique (Table 1) [34–37]. It was found that the mtDNA content is significantly associated with clinical outcomes, in which low tumor mtDNA content is associated with worse survival in adrenocortical carcinoma and low-grade glioma [35]. Importantly, these comprehensive studies confirmed the previous findings that most tumors (over 50%) carry somatic mtDNA mutations [37, 38].

**Table 1:** The mitochondrial DNA mutations in cancers with the next-generation sequence (NGS) assay

Ju, et al. Elife. 2014;3:e02935	Grandhi, et al. Hum Mol Genet. 2017;26:2912–2922	Yuan, et al. Nat Genet. 2020;52:342–352
TCGA; 704 WGS; 971 WES; 31 tumor types	TCGA, 1,916 NGS; 24 tumor types	ICGC/TCGA-PCAWGC; 2,658 WGS; 38 tumor types
✓ Among 1675 cancer samples, 976 (58.3%) harbor at least one somatic substitution and 521 (31.1%) carry multiple substitutions, ranging from 2 to 7	✓ 64.3% of cancers harbor somatic mtDNA mutations	✓ Over 85% of somatic mtDNA substitutions are heteroplasmic
✓ Gastric, hepatocellular, prostate, and colorectal cancers are detected with the highest number of mtDNA substitutions. Hematologic cancers (acute lymphoblastic leukemia, myeloproliferative disease, and myelodysplastic syndrome) harbor fewer mutations	✓ Metastatic and recurrent tumors harbor a larger proportion of RNA variants vs. D-loop/unannotated variants than primary tumors	✓ MtDNA mutations are largely proportional to age
✓ Among the 1907 substitutions, 1153 (60.5%) are in the 13 protein-coding genes	✓ Higher numbers of tRNA, rRNA, and mRNA aberrations are detected in tumors than in normal cells ✓ Several recurrent mtDNA mutations occur within the genes of NADH dehydrogenase complex (Complex I)	✓ Variant allele fraction analysis showed that mutational hotspots are in the D-loop region and ND4 gene ✓ ND5 gene is the most frequently mutated in most cancer types; ND4 gene is the most frequently mutated in prostate and lung cancers; COX1 gene is the most frequently mutated in breast, cervical, and bladder cancers
✓ The vast majority of mtDNA mutations are passengers with no convincing evidence suggesting the existence of driver mitochondrial DNA mutations ✓ Mutations in tRNA anticodons and protein-truncating mutations confer a selective disadvantage ✓ No evidence of the mutational signatures characteristic of these carcinogens among the mtDNA mutations could be found	✓ There is no evidence for positive or negative selection for the somatic mtDNA mutations, except for KICH and thyroid carcinoma (dN/dS results most consistent with positive selection) ✓ Non-synonymous substitutions below 5% in the normal cell expand to a median allelic frequency of 58.8% in the tumor, as compared to 18.8% for synonymous substitutions, suggesting positive selection in the tumor	✓ Truncating mutations might be a negative selection

*KICH* kidney chromophobe, *KIRC* kidney renal clear cell carcinoma, *KIRP* kidney renal papillary cell carcinoma

Whether mtDNA mutations are driver mutations or passenger alterations in carcinogenesis is still controversial [39]. The nonsynonymous (changed amino acid sequence) to synonymous (identical amino acid) mutation (dN/dS) ratio of the mtDNA mutations in malignancies was significantly higher than the random expectation, suggesting that the high dN/dS ratio of mtDNA mutations has a positive selection advantage [40]. Moreover, it was suggested that there is weak positive selection for missense mutations in mtDNA and neutral selection for nonsense mutations in mtDNA in cancers [36]. Most somatic mtDNA mutations in cancer were found to be heteroplasmic [37]. Heteroplasmy in tumors with frameshift mutations (mono- or di-nucleotide insertions or deletions) and nonsense mutations of mtDNA is lower than that in tumors with silent mutations of mtDNA, while heteroplasmy in tumors with missense mutations of mtDNA is higher than that in tumors with silent mutations of mtDNA [36, 37, 41]. These results suggested that destructive mtDNA mutations might contribute to negative selection in cancer. However, another analysis revealed that protein-truncating mtDNA mutations, predominantly located on Complex I genes, might contribute to positive selection in specific cancers such as colorectal, kidney, and thyroid cancers [38]. These findings suggested that tissue types, affected genes, and mutant types might contribute to different selections for mtDNA mutations in cancers.

Somatic mutations or reduced mtDNA copy numbers in cancer cells might lead to mitochondrial dysfunction and might be implicated in cancer progression to malignancy. Several studies using cybrid cell models revealed that a pathogenic T8993G mutation in the mtDNA ATP synthase subunit 6 gene promotes tumor growth in nude mice by enhancing ROS production and preventing apoptosis [30, 42, 43]. Another cybrid cell study showed that a 12418insA mutation in the mtDNA NADH dehydrogenase subunit 5 gene that causes a frameshift and truncated protein of the ND5 subunit might lead to reduced OXPHOS function and increased ROS production in human cancer cells and enhanced tumor growth in nude mice [44]. Moreover, ROS-generating mtDNA mutation-mediated mitochondrial dysfunction was reported to contribute to metastatic cancer phenotypes [45]. These lines of evidence support that somatic mutations in the protein-coding region of mtDNA might contribute to tumor growth and cancer progression to malignancy.

It was found that osteosarcoma 143B cells with mtDNA depletion (ρ^0^) or cybrids harboring severely damaged mitochondria did not produce tumors, whereas cybrids containing mild mtDNA mutations with impaired OXPHOS could enhance tumorigenesis [46]. Similarly, mild mtDNA deletion might increase mitochondrial biogenesis and enhance proliferation, promoting tumor growth of Hodgkin and Reed-Sternberg cells [47]. Therefore, the role of mtDNA alterations in cancer progression might depend on tissue types, affected genes, and mutant types [48]. Further understanding of the mtDNA mutation-mediated selective pressures in cancer progression might need a detailed study with a single-cell analysis technique.

### Mitochondrial fission might enhance cancer progression

Mitochondria are highly dynamic organelles that undergo continuous fusion and fission [49]. Mitochondrial fusion expands mitochondrial connections and increases the capacity of OXPHOS for energy needs. Moreover, the fusion of damaged and healthy mitochondria might mitigate metabolic stress, decrease mitochondrial fragmentation and maintain mitochondrial function. Mitochondrial fusion involves outer membrane fusion with mitofusin (MFN) 1 and MFN2 and inner membrane fusion with optic atrophy 1 (OPA1). In addition, mitochondrial phospholipase D activates GTPases to fuse mitochondrial membranes [50]. On the other hand, mitochondrial fission is a sequential process coordinated with various factors, such as dynamin-related protein 1 (DRP1, recruited to the outer membrane and complexed with mitochondria), mitochondrial fission factor, mitochondrial fission protein 1, mitochondrial dynamic protein of 49 kD, and mitochondrial dynamic protein of 51 kD [51].

Mitochondrial dynamics are tightly controlled by the needs of cellular metabolism and mitochondrial function [52]. Mitochondrial dynamics might contribute to the dilution of mtDNA mutation- and oxidized protein-induced cellular stress [53]. In addition, mitochondrial dysfunction-mediated mitochondrial fission might contribute to DNA damage, which is caspase-dependent [54]. In mtDNA-depleted C2C12 cells, OPA1 is decreased with increased DRP1 expression [52]. In addition, mtDNA depletion-mediated metastatic potential might originate from high mitochondrial fission [52]. On the other hand, oncogenic Kirsten rat sarcoma viral oncogene homolog (KRAS) mutation in pancreatic cancer might promote mitochondrial division and activation of DRP1, which are essential to KRAS-driven cancer progression [55, 56].

In most lines of evidence, mitochondrial fission might contribute to poor prognosis for various cancers. In renal clear cell carcinoma, low MFN2 expression was found to be associated with a poor prognosis [57]. DRP1 was also found to be activated in brain tumor-initiating cells and related to poor prognosis in glioblastoma [58]. In hepatocellular carcinoma, downregulated OPA1-mediated mitochondrial dysfunction might result in aberrant mitochondrial fission and promote cancer cell growth by metabolism remodeling [59]. DRP1 coexpressed with cell cycle-related genes was also found to promote the proliferation of ovarian cancer cells [60]. DRP1-mediated mitochondrial fission might also promote cell migration in hepatocellular carcinoma (HCC) [61]. Moreover, MFN1 loss might induce the epithelial-to-mesenchymal transition (EMT) of HCC cells [62]. DRP1-mediated mitochondrial fission was also found to be crucial for cisplatin resistance and to be associated with the Warburg effect in ovarian cancer cells [63, 64]. Furthermore, DRP1-mediated mitochondrial fission might contribute to the survival and chemoresistance of breast cancer cells [65, 66]. These findings suggest that mitochondrial fission might enhance the proliferation, metastasis, and chemoresistance of cancer cells, thus promoting cancer progression to malignancy.

### Mitochondrial retrograde signaling pathways might promote cancer progression and potentially be a valuable therapeutic target

Several lines of evidence suggest that mitochondria are crucial information processors in cells and can interconnect with the nucleus and other organelles [67]. It was recently proposed that mitochondria can sense, integrate, and derive signaling in the mitochondrial information processing system [67]. Mitochondrial retrograde signaling, a mitochondria-to-nucleus communication, was initially proposed in a yeast model [68]. The signaling pathway acts as a homeostatic or stress response mechanism to adjust metabolic activities in response to various changes in mitochondrial function.

In humans, mitochondrial retrograde signaling has been proposed to be involved in several pathologic diseases, such as osteoarthritis, Alzheimer's disease, and cancer [32, 33, 69, 70]. Several mitochondrial retrograde signaling pathways, including several mitochondrion-derived molecules (ROS, calcium, oncometabolites, exported mtDNA, mitochondrial double-stranded RNA, humanin, and MOTS-c), the mitochondrial unfolded protein response (mtUPR), and the integrated stress response (ISR), have been implicated in the progression of cancer cells to malignancy [33]. Targeting mitochondrial retrograde signaling might be a potential therapeutics against cancer progression.

### Mitochondrial retrograde signaling pathways contribute to tumor formation and cancer cell proliferation

ROS, the byproducts of mitochondrial respiration, are important mediators of mitochondrial retrograde signaling. Succinate dehydrogenase (SDH)-B inhibition-elevated ROS were found to activate HIF-1α and consequently contribute to tumor formation in hepatoblastoma, HCC, lung carcinoma, and osteosarcoma cells [71]. In addition, the mutated SDH-C-increased ROS levels might contribute to DNA mutations and tumor formation in a transgenic mouse model [72]. miR-663, a tumor suppressor gene-like function that might influence arsenic-induced skin carcinogenesis by mitochondrial alterations [73], is downregulated in mtDNA-depleted ρ^0^ cells [74]. ROS were found to be crucial for the epigenetic regulation of miR-663, and the decreased miR-663 expression might promote tumor development in vivo in mice and be a poor prognosis factor for breast cancer patients [74].

Calcium/calcineurin-mediated mitochondrial retrograde signaling might be vital to tumorigenesis [75]. MtDNA depletion caused by loss of transcription factor A, mitochondrial, might affect tumor cell differentiation and proliferation through the calcium-CFAP65-phosphoenolpyruvate carboxykinase 1 axis [76]. Moreover, cytochrome c oxidase defects might lead to increased glycolysis and carcinogenesis via calcium/calcineurin-PI3K signaling [77].

DRP1-mediated mitochondrial fission increased cytosolic mtDNA stress and further enhanced chemokine (C–C motif) ligand 2 secretion from HCC cells by the TLR9-NF-κB signaling pathway, which might result in tumor-associated macrophage-mediated tumor growth [61].

Several oncometabolites, such as 2-hydroxyglutarate (2HG), succinate, and fumarate, accumulate due to mutations in nuclear-encoded mitochondrial enzyme genes, including isocitrate dehydrogenase (IDH) 1 and 2, SDH, and fumarate hydratase (FH), in human cancers [78]. 2HG facilitates epigenetic regulation by interfering with α-KG-dependent dioxygenases, such as cytosine hydroxylases and histone demethylases [79]. In addition, 2HG inhibits ten-eleven translocation (TET) methylcytosine dioxygenase 2 and DNA hypermethylation, which might be crucial for IDH mutation-related tumorigenesis [80]. These results suggest that 2HG might be an oncogenic driver through epigenetic regulation. On the other hand, 2HG might activate hypoxia signaling through upregulation of HIF-1α by inhibition of prolyl-hydroxylase domain (PHD)-mediated proteasomal degradation [81]. Similar to 2HG, the accumulation of succinate and fumarate might inhibit TET enzymes and PHD, which might elevate HIF-1α signaling and driven tumorigenesis [82, 83].

### Mitochondrial retrograde signaling pathways enhance cell migration, invasion, metastasis, and angiogenesis

Mitochondrial dysfunction-increased ROS might upregulate amphiregulin to promote cell migration of hepatoma cells [84]. ROS-heat shock factor 1-claudin-1 was found to be involved in mitochondrial dysfunction enhanced invasiveness of hepatoma cells [85]. In addition, mitochondrial ROS-mediated EMT signaling pathways were found in various cancer cell models. Downregulation of TMEM126A, a mitochondrial transmembrane protein, was shown to contribute to breast cancer metastasis via ROS-EMT signaling [86]. A defect in mitochondrial nucleoside diphosphate kinase might promote EMT, migration, and invasion via metabolism remodeling and ROS production [87]. Mitochondrial respiratory defects might enhance hepatoma cell invasiveness via the ROS-mediated signal transducer and activator of transcription 3 (STAT3)-NFE2L1-STX12-EMT axis [88].

It was found that mtDNA loss in human mammary epithelial cells could activate calcineurin-dependent EMT-like reprogramming to migratory and invasive phenotypes [89]. Mitochondrial dysfunction might also contribute to liver cancer cell invasion via calcium-nuclear protein 1-granulin signaling [90]. Moreover, downregulation of single-strand DNA-binding protein 1 in highly metastatic breast cancer cells might decrease mtDNA copy number and contribute to triple-negative breast cancer (TNBC) metastasis via the calcineurin-c-Rel/p50 nuclear localization-transforming growth factor-β-EMT pathway [91].

It was also found that succinate may promote cancer metastasis in non-small cell lung cancer cells through the succinate receptor SUCNR1-PI3K/Akt-HIF-1α pathway [92]. Similar to SDH mutation, FH deficiency in tumors might also result in an accumulation of fumarate and succinate, which contribute to increased HIF1-α levels and angiogenesis [93]. In addition, fumarate was found to be involved in endometrial cancer cell proliferation, migration, and invasion via elevated adenylosuccinate lyase-fumarate-killer cell lectin-like receptor C3 signaling [94].

MtUPR is an emerging mitochondrial retrograde signaling pathway that alleviates the harsh tumor microenvironment in cancer cells [95]. The mtUPR is a mitochondrial stress response responsible for protein homeostasis by increasing nuclear gene expression of mitochondrial heat shock proteins and proteases [33]. In mammalian cells, C/EBP homologous protein, activating transcription factor 4 (ATF4), and activating transcription factor 5, which are homologs of ATFS-1 in the *C. elegans* model, are crucial for the activation of mtUPR. Recently, ISR was proposed to be involved in mitochondrial retrograde signaling [96]. The eukaryotic translation initiation factor 2α (eIF2α) is the main component of ISR. Four eIF2α kinases have been identified, including general control nonderepressible 2 (GCN2, activated by amino acid deprivation), protein kinase R-like endoplasmic reticulum kinase (PERK, triggered by endoplasmic reticulum stress), heme-regulated inhibitor kinase (HRI, activated by ROS, heme deficiency, and osmotic and heat shock), and protein kinase R (PKR, triggered by double-stranded RNA, e.g., viral infection). PERK, GCN2, and HRI are activated by various types of mitochondrial stress [33]. The ISR confers eukaryotic cells with the adaptive ability to restore cellular homeostasis under various stress conditions [97]. The phosphorylation of eIF2α at serine51 reduces global protein synthesis but increases the protein translation of some stress response genes with several small upstream open-reading frames (ORFs) in the cis-regulatory elements of the 5’-UTR region and internal ribosome entry sites [98]. ATF4, a crucial mediator of mtUPR and ISR, contains upstream ORFs and is selected for translation under stressful circumstances. ATF4 is translocated into the nucleus and increases the gene transcription of several pro-survival factors, including antioxidant enzymes, transport and biosynthesis of amino acids, and autophagy [99].

In breast cancer cells, the mitohormesis-mediated mtUPR is important to the invasiveness and metastasis of cancer cells, and high expression of the 7-gene mtUPR signature might contribute to poor clinical outcomes [100]. Moreover, human epidermal growth factor receptor 2 or starvation might promote cell metastasis in nontransformed mammary epithelial cells and cancer cells via the ISR pathway [101, 102]. These findings suggest that targeting the mtUPR or ISR might be a promising therapeutic strategy against cancer progression.

### Mitochondrial retrograde signaling pathways promote therapy resistance

Mitochondrial dysfunction-mediated ROS and upregulated amphiregulin might induce drug resistance to endocrine therapy through amphiregulin-estrogen receptor loop signaling in hormone receptor-positive breast cancer cells [103]. In addition, mitochondrial dysfunction-increased ROS upregulates amphiregulin to promote chemoresistance in hepatoma cells [84].

Calcium was also found to be involved in mitochondrial dysfunction-mediated amphiregulin upregulation, which contributes to chemoresistance and cell migration of hepatoma cells [84], and drug resistance to endocrine therapy in hormone receptor-positive breast cancer cells [103]. Recently, mitochondrial dysfunction was found to induce radioresistance of colorectal cancers by calcium-PDP1-pyruvate dehydrogenase-histone acetylation regulation and to enhance the DNA damage repair response [104]. In addition, oxidative damage of mtDNA-upregulated Lon might contribute to cisplatin resistance by mitochondrial Na^+^/Ca^2+^ exchanger NCLX-mediated calcium-PYK2-SRC-STAT3-interleukin (IL) 6 signaling [105].

2HG can interfere with the association between Cdc42 and MLK3, which might result in apoptosis resistance and promote cancer cell proliferation [106]. In addition, 2HG might inhibit cytochrome c release and increase anti-apoptotic bcl-2, contributing to cancer progression [107]. Moreover, 2HG was found to induce resistance to histone deacetylase inhibitors by NANOG-mediated multidrug resistance protein 1 expression [108]. Furthermore, succinate might be linked with chemoresistance through HIF-1α-mediated drug efflux transporters, such as P-glycoprotein, multidrug resistance-related protein 1, and breast cancer resistance protein [109].

Cytosolic mtDNA molecules might contribute to cancer chemoresistance by engaging innate immune nucleic acid sensors, upregulating interferon-stimulated genes, and resulting in an elevation of nuclear DNA repair [110].

Disruption of mitochondrial Lonp1 protease, responsible for mitochondrial quality control, can activate the ISR, mtUPR, cytosolic UPR, and redox homeostasis against anticancer therapy via induction of adaptive cytoprotective mechanisms [111]. MtDNA alterations in cancers might activate the mtUPR [112]. The mtUPR in cancer might support the mitohormetic zone to induce cancer cells to adapt to oxidative stress through superoxide dismutase type 1 and 2 [112]. Several ATF4 downstream targets, such as xCT, a glutamine-cysteine antiporter involved in the x_c_^−^ system for supporting cellular glutathione synthesis, have been found to serve as mediators of cancer progression [33]. The x_c_^−^ system, composed of xCT (light-chain) and 4F2 heavy chain, is responsible for cysteine/glutamate exchange and crucial for cell growth, metastasis, and chemoresistance in several cancer cells [113]. It was further demonstrated that mitochondrial dysfunction can enhance the chemoresistance of cancer cells via the GCN2-eIF2α-ATF4-xCT pathway [114].

### Mitochondrial retrograde signaling pathways induce immune evasion

ROS might contribute to immune escape in the tumor microenvironment [115]. Evidence revealed that ROS might damage mtDNA and contribute to immune escape by the stimulator of interferon genes (STING)-interferon-programmed cell death 1-ligand 1 (PD-L1) signaling [116]. Additionally, ROS might enhance the secretion of NF-κB-dependent inflammatory cytokines to suppress the antitumor function of macrophages, dendritic cells, and T cells [115]. Therefore, the modulation of ROS in the tumor microenvironment might have translational and clinical significance in boosting the efficacy of cancer immunotherapy [117]. However, ROS might contribute to the enhancement of antigen presentation, upregulating the immune response, and decreasing immune escape, and ROS-responsive prodrugs might elevate the efficacy of cancer treatment [118, 119]. The exact role of ROS modulators in cancer immunotherapy warrants further investigation.

On the other hand, recent evidence suggested that mitochondrial stress in the tumor microenvironment might contribute to cancer immune escape [115]. MtDNA might be released from mitochondria under oxidative stress [120]. Circulating mtDNA molecules released by cancer cells may inhibit leukocytes from producing inflammatory cytokines, such as IL-6 and tumor necrosis factor-α, which might result in immune escape [121]. In addition, the horizontal transfer of mtDNA from cancer cells to immune cells might deactivate the immune response by inducing apoptosis events in immune cells [121]. Moreover, upregulation of mitochondrial Lon might induce oxidized mtDNA release into the cytosol and contribute to immune escape through stimulator of interferon genes-tank-binding kinase-interferon signaling-elevated PD-L1 and indoleamine 2,3-dioxygenase 1 [116].

However, other evidence revealed that cancer immunotherapy targeting CD47 can induce cancer cells to leak mtDNA into nearby dendritic cells and present antigens to effector T cells, bridging the innate and adaptive immune systems [122]. Moreover, inhibition of ataxia telangiectasia mutated protein might boost the efficacy of anti-programmed death-1 (PD-1) therapy by promoting mtDNA leakage and cGAS/STING activation [123]. The role of released mtDNA in cancer immunotherapy remains to be further investigated.

### Targeting mitochondrial retrograde signaling might be a promising strategy for treating cancer malignant progression

Antioxidants, such as N-acetylcysteine (NAC), can suppress mitochondrial dysfunction-induced cell migration in human gastric cancer cells [124, 125]. In addition, NAC can counteract mitochondrial dysfunction-enhanced amphiregulin, which is associated with chemoresistance and cell migration in HepG2 cells [84]. Moreover, NAC can reverse mitochondrial dysfunction-mediated endocrine therapy resistance [103]. Targeting ROS in mitochondrial retrograde signaling might be a potential option against mitochondrial dysfunction-mediated cancer progression. However, recent evidence proposed that mitochondria-targeted antioxidants, such as MitoQ and MitoTEMPO, could not significantly alter the progression of v-raf murine sarcoma viral oncogene homolog B1-induced melanoma and KRAS-induced lung cancer in endogenous animal models [126]. Further studies are warranted to reveal the role of mitochondrial ROS in cancer progression.

Downregulation of calcium signaling with BAPTA-AM, a calcium-chelating agent, can counteract mitochondrial dysfunction-mediated amphiregulin, which is responsible for chemoresistance and cell migration in HepG2 cells [84]. In addition, BAPTA-AM can mitigate mitochondrial dysfunction-enhanced endocrine therapy resistance [103]. Antiresorptive agents, such as bisphosphonates and denosumab, have clinical benefits for osteoporosis, malignant hypercalcemia, and bone metastasis. Additionally, adjuvant bisphosphonate therapy with endocrine therapy might improve the disease-free survival of patients with early breast cancer [127]. A current meta-analysis further demonstrated that early usage of antiresorptive agents could decrease endocrine therapy resistance in early breast cancer patients with adjuvant endocrine therapy [128]. Moreover, several calcium channel blockers, such as verapamil, nifedipine, diltiazem, and amlodipine, might be used against cancer [129]. Ca^2+^ can be taken up into mitochondria by the mitochondrial calcium uniporter (MCU) and released into the cytosol by the mitochondrial Na^+^/Ca^2+^ exchanger [130]. Furthermore, Ru360, a selective MCU inhibitor, might slow the metalloprotease-processed CD95L-enhanced cell migration of BT549 TNBC cells [131]. Targeting calcium-mediated mitochondrial retrograde signaling in clinical practice might be a potential therapeutic strategy against cancer progression.

Some specific types of cancer might depend on xCT, eliciting the progression of cancer stem cells and crosstalk between xCT and tumor immunity [132]. Hence, targeting xCT might be a potential therapeutic strategy against cancer progression. Recently, a preliminary clinical trial was initiated; however, a further issue about the administration of xCT inhibitors remains to be addressed [133]. On the other hand, upregulated xCT might contribute to metabolic reprogramming and glucose dependence for cancer cell survival [134]. In addition, ISR-upregulated xCT enhanced cancer cell death under glucose starvation via mitochondrial ROS [135]. Hence, the glycolytic inhibitor might have a selective antitumor effect in xCT-high-expressing cancers due to the decreased metabolic flexibility. Moreover, combining ISR activators, such as salubrinal and nelfinavir, with glycolysis pathway inhibitors (including rapamycin, ritonavir, and metformin) might be a reasonable strategy against cancer progression [135]. Furthermore, high xCT-expressing cancer cells might be glutamine or cystine dependent [136]. Some lines of evidence further reveal that CB-839, a glutaminase inhibitor, or cyst(e)inase might be used against cancer [137, 138]. Targeting glutamine or cysteine metabolism might also be a reasonable strategy against cancers with elevated xCT, such as TNBC or pancreatic ductal adenocarcinoma cancer cells [139, 140].

Growth differentiation factor 15 (GDF15), one of the mitokines, can communicate mitochondrial stress to the adaptation response in the physiologic process [141]. GDF15 might be implicated in cancer progression and is also regulated by ISR [33]. Recent evidence suggests that GDF15 might be essential for cell proliferation, cell migration, and cisplatin resistance in human gastric cancer [142]. In addition, GDF15 might be a promising targetable immune checkpoint because it can inhibit dendritic cell maturation and immune cell infiltration [143]. A Phase 1 trial of the GDF15 neutralizing antibody, CTL-002, is currently being conducted for its use against advanced-stage solid tumors (NCT04725474, ClinicalTrials.gov) [144]. Targeting ISR-GDF15-mediated mitochondrial retrograde signaling might be a possible treatment modality against cancer progression.Recently, ghost mitochondria in cancer were proposed to address the controversy of mitochondrial reprogramming as a tumor driver [25]. The dysregulation of Mic60, a component of the multiprotein mitochondrial inner membrane complex and responsible for maintaining cristae, respiratory complexes, and outer membrane biogenesis, was observed in various types of cancer [145]. Decreased Mic60 expression might slow cancer cell proliferation but enhance inflammation, cell quiescence, mitochondrial dynamics, cancer cell invasion, and metastasis through several mitochondrial retrograde signaling pathways, such as ROS, ATP, the type I interferon/senescence-associated secretory phenotype transcriptional signature, and the GCN2-ISR pathway [25]. Hence, the pharmacologic targeting of GCN2, an ATF4 upstream regulator, might have benefits against Mic60-low-expressing cancer [145].

### Role of mitochondria in cancer immunity

Recent evidence emphasized that mitochondria are essential for cancer immunity. The regulation of the tumor microenvironment, involving cancer cells and immune cells, plays a vital role in cancer immunity [146]. Hence, some obstacles in canonical mitochondrial-specific anticancer strategies targeting cancer cells are raised because they might adversely affect cancer immunity. Recently, mitochondrial-related gene signature was proposed as the tumor immune microenvironment evaluation, including immune cell infiltration [147]. Understanding how mitochondria affect the immune system is a well-concerned topic in cancer research.

### Mitochondria are involved in the regulation of T cells

In the immune system, mitochondria-produced ATP is essential for the proliferation, differentiation, and activation of immune cells [148, 149]. Different immune cells have specific metabolic demands and signaling pathways to support their biological processes. During the maturation of T cells, quiescent naïve T cells that rely on OXPHOS to produce energy are differentiated into effector T cells that depend on glycolysis. Metabolic remodeling from OXPHOS to glycolysis supports T-cell proliferation and provides metabolic intermediates via several pathways, such as HIF-1α- or MYC-activated PI3K/AKT/mTOR [150, 151]. In addition, mitochondrial ROS might activate T cells and promote antigen-specific proliferation [149]. Moreover, ROS are essential for the T-cell immune response in the tumor microenvironment [152], which might be crucial for the immune response and antitumor function. In contrast, regulatory and memory T cells are dependent on OXPHOS and fatty acid oxidation for survival and differentiation [153]. Effector T cells become memory CD8 T cells with enhanced fatty acid oxidation by elevated AMP-activated protein kinase (AMPK) or inhibited mTOR pathways during the contraction phase of T cells (effector to memory transition) [154].

In addition to metabolic remodeling, mitochondrial dynamics are important for T-cell reprogramming. Activation of T cells by T-cell receptors is remodeled to aerobic glycolysis and associated with enhanced mitochondrial fission, increased mitochondrial number, and flabby cristae [155]. The flabby mitochondrial cristae might decrease ETC efficiency, increase aerobic glycolysis, and raise ROS levels [155]. On the other hand, mitochondria fuse with intact cristae and undergo functional OXPHOS when recovered from effector T cells to memory T cells [155]. Memory T cells have higher mitochondrial respiratory capacity than effector T cells for preserving the longevity of memory T cells [156].

### Mitochondria are involved in the regulation of macrophages and nature killer (NK) cells

Mitochondria play vital roles in the differentiation and activity of macrophages. Proinflammatory macrophages (M1 subtype) are differentiated by lipopolysaccharide with a metabolic remodeling shift from OXPHOS to aerobic glycolysis and increased succinate and mitochondrial ROS levels [157]. On the other hand, anti-inflammatory macrophages (M2 subtype) are differentiated by IL-4 with increased OXPHOS and fatty acid oxidation [158, 159]. Treatments with chloroquine, which remodels the metabolism from OXPHOS to glycolysis, might inhibit tumor formation via induction of the proinflammatory M1 phenotype [160].

Mitochondrial dynamics might be necessary for macrophage differentiation. M1 and M2b (Th2 activation, immunoregulation) macrophages have highly fragmented mitochondria [161, 162]. In contrast, M2a (Th2 responses, anti-inflammatory) and M2c (immunoregulation, tissue remodeling, efferocytosis) macrophages have elongated and connected mitochondria and rely on OXPHOS via increased mitochondrial fusion [161, 162].

On the other hand, NK cells, cytotoxic lymphocytes in the innate system, mainly utilize glucose through elevated glycolysis and OXPHOS to support cytokine secretion and cytotoxic activity [163]. The mitochondrial receptor BNIP3–BNIP3L protein might promote the generation of natural killer cell memory via mitophagy-removed damaged mitochondria and reduced oxidative stress [164].

### Tumor microenvironment and metabolism remodeling are crucial for cancer immunity

Cancer cells and tumor-infiltrating lymphocytes (TILs) compete with each other in the tumor microenvironment for glucose and other nutrient demands. Cancer cells, which prefer aerobic glycolysis, might limit the glucose utilization of TILs and cause TILs to rely on OXPHOS. Moreover, the limited nutrients and oxygen in the tumor microenvironment might suppress the mitochondrial function of TILs [165]. In addition, cancer-mediated metabolic stress might cause CD8 + TILs exhaustion [166]. The induced metabolic remodeling from glycolysis to fatty acid oxidation might be used to preserve the functions of CD8 + TILs [166]. Moreover, intolerant oxidative stress might be involved in T-cell exhaustion via the nuclear factor of activated T-cell activation [167, 168]. The morphology of mitochondria in tumor-infiltrated NK cells shows fragmented status, which might be originated from a hypoxic condition in the tumor microenvironment, and might contribute to reducing the tumoricidal ability of NK cells [169]. Furthermore, hypoxia and HIF-1α are associated with downregulating major histocompatibility complex (MHC)-I, a significant antigen presentation pathway [170]. On the other hand, cytosolic mtDNA might enhance tumor cell immunogenicity [171]. MtDNA alteration, such as alternative mitochondrial cytochrome b, might elevate CD4 + T-cell response [172]. However, the IDH mutant glioma cells might escape NK cell immune surveillance by downregulating NKG2D ligand expression [173].

Metabolic stress might stimulate the PD-1 or lymphocyte-activation gene 3 (LAG-3)-immune suppressive checkpoint pathways [166]. In addition, increased aerobic glycolysis in cancer cells might promote the expression of PD-1 and LAG-3 in TILs and reduce mitochondrial mass and glucose uptake [174]. Moreover, HIF-dependent vascular endothelial growth factor might enhance PD-1 expression in tumor-infiltrating CD8 + T cells [175]. On the other hand, PD-1, PD-L1, and cytotoxic T-lymphocyte-associated protein 4 (CTLA-4) might modulate the metabolic functions of cancer and infiltrating immune cells [176–178]. In addition, the number and length of mitochondrial cristae would be decreased by PD-1 activation, emphasizing the mitochondria function and structure in memory T cells [179].

Emerging evidence has suggested that the mitochondrial dynamic change might contribute to immune escape via decreased tumor immunogenic antigens, which might result in the decreased cytotoxic activity of T cells [180]. Mitochondrial fission can downregulate the expression of MHC-I antigens and contribute to the weak immunogenicity of cancer cells [180]. However, mitochondrial fission might enhance lymphocyte chemotaxis and cancer cell migration [181, 182]. Additionally, inhibition of the E26 transformation-specific transcription factor ELK3 might contribute to the restorative effect of NK cells against TNBC cells through mitochondrial fission-mediated superoxide accumulation [183].

It was recently proposed that cancer cells might be able to hijack mitochondria from nontumor cells in the tumor microenvironment [184, 185]. Mitochondrial transfer is one type of intercell communication [186]. Cancer cells might obtain mitochondria from T cells via nanotubes to strengthen cancer cells, resulting in immune escape [187]. These findings suggest that investigating the roles of mitochondria and metabolic remodeling of cancer and immune cells in the tumor microenvironment is important to develop antitumor immunotherapies [188].

### Targeting mitochondria and metabolism in cancer immunotherapy

Antibodies against PD-1, PD-L1, and CTLA-4 have been introduced against various types of cancers by counteracting immune checkpoints and enhancing immune attacks. However, many cancer patients do not respond to these immune checkpoint inhibitors. Recently, several lines of evidence have supported that immune checkpoints might be involved in the regulation of cell metabolism and mitochondria in immune cells or cancer cells, and drug combination with mitochondrial modulating agents could enhance the therapeutic efficacy of these immune checkpoint inhibitors.

PD-1 signaling decreases glycolysis and increases fatty acid oxidation in T cells via the elevation of CPT1A expression [179]. CTLA-4 signaling might inhibit glycolysis in immune cells [179]. Interestingly, mitochondrial activation might augment the efficacy of anti-PD-1 therapy [178]. Moreover, evidence has shown that PD-L1 signaling upregulates glycolysis via the AKT-mTOR pathway in cancer cells [189]. Anti-PD-L1 therapy might suppress glycolysis and thus repress cancer progression [189, 190]. A preclinical study [178] showed that combinations of the activators of mTOR, AMPK, or peroxisome proliferator-activated receptor-gamma coactivator-1α (PGC-1α) might synergistically enhance the anticancer effects of anti-PD-1 treatment [178]. It was also found that PGC-1α can mitigate T-cell exhaustion [176]. Similarly, bezafibrate, an agonist of PGC-1α/PPAR complexes, might elevate the number of TILs and the efficacy of anti-PD-1 therapy [191]. In addition, spermidine, a biogenic polyamine that can activate fatty acid oxidation activity and enhance OXPHOS, was proposed to increase the effectiveness of anti-PD-1 therapy [192]. In contrast, high-OXPHOS might be a barrier to ant-PD-1 therapy in melanoma [193]. Combining radiotherapy and OXPHOS inhibitor with immunotherapy therapy might enhance the anticancer effect and reduce anti-PD-1 resistance [194, 195]. Moreover, mitochondria-targeting polymer micelle might enhance the effectiveness of anti-PD-L1 therapy in osteosarcoma [196]. These findings suggest that the combination of mitochondrial modulators and immunotherapy might provide a therapeutic strategy for cancer immunotherapy nonresponders. However, future study warrants further investigation on how to practically apply specific drugs regulating OXPHOS, metabolic pathway, or mitochondrial dynamics between tumor and immune cells or different stages and types of immune cells.

Currently, reliable predictive biomarkers for cancer immunotherapy warrant further investigation. The mitochondrial role in the efficacy of cancer immunotherapy has recently been a concern [197]. A correlation between the mitochondrial characteristics of patients and the response to anti-PD-1 immunotherapy might be observed. Mitochondria-related factors (such as age, male sex, smoking, obesity, and exercise insufficiency) might be associated with PD-1 expression in T cells, potentially elevating the clinical benefits of cancer immunotherapy. In the KEYNOTE-181 and ATTRACTION-3 trials, anti-PD-1 monotherapy had a better clinical response in the subgroups of male sex and younger age [198, 199]. The young age factor might be linked to high mitochondrial biogenesis, while the male sex factor might be related to PD-1 expression [197]. In addition, the Asian subgroup might have a good prognosis for anti-PD-1 therapy [198, 199], which might originate from the higher oxidative capacity of mitochondria in Asian patients than in white patients [200]. Moreover, the efficacy of front-line cancer immunotherapy is better than that of later-line treatment [201], which might originate from the negative effect of anticancer therapy on mitochondria. Understanding the mitochondrial role in cancer immunotherapy might contribute to personalized and precision treatment for elevating the efficacy of cancer immunotherapy.

Targeting metabolism or mitochondria might be a reasonable strategy to enhance cancer immunotherapy. Evidence has shown that anti-CTLA-4 therapy might decrease Treg stability in low-glycolytic tumors [202]. In addition, the inhibition of glycolysis might elevate the antitumor effect of effector T cells and NK cells [203]. Moreover, 2-deoxyglucose targeting glycolysis might inhibit the effects of myeloid-derived suppressor cells in the tumor microenvironment and enhance anticancer immunity in TNBC [204]. On the other hand, hypoxia, a well-known characteristic of the tumor environment, is harmful to the antitumor function of the immune system [205]. In addition, metformin-normalized tumor hypoxia might enhance the efficacy of anti-PD-1 therapy [206]. Therefore, a phase II clinical trial of anti-PD-1 mAb therapy alone or with metabolic modulators to reverse tumor hypoxia and immune dysfunction in solid tumor malignancies is recruiting participants (NCT04114136, ClinicalTrials.gov). Lactate-mediated acidity in the tumor microenvironment might be a target for cancer immunotherapy. Neutralizing acidity by bicarbonate might boost the efficacy of anti-CTLA-4 and anti-PD-1 antibodies [207]. In addition, AZD3965, which inhibits monocarboxylate transporter 1 and decreases lactate utilization, might induce T cells and decrease tumor-promoting M2 macrophage polarization [208]. Targeting other mitochondrial pathways might improve cancer immunity. Venetoclax, a clinical hematologic malignancy medication inhibiting mitochondrial anti-apoptotic bcl2, was proposed to enhance NK cell-mediated anticancer effect [209]. Moreover, L-778123, a dual farnesyltransferase and geranylgeranyltransferase inhibitor, could sensitize the antitumor outcomes of anti-PD-L1 in breast cancer animal models via inhibition of mitochondrial hijacking [187]. Moreover, promoting mitochondrial fusion drugs, such as fusion promoter M1 and mdivi-1, might prolong the effect of CD8 + T cells and improves cellular immunotherapy against tumors [155]. In addition, Fe^2+^–Ru^2+^-loaded mesoporous silica nanoparticles, inducing mtDNA oxidative damage in cancer cells, might polarize to M1 tumor-associated macrophages and activate the immune response of macrophages against cancer through oxidative mtDNA [210].

Recently, COX1 mutant-derived tumor mitochondria vaccine was proposed as a potential therapeutics against renal cell carcinoma via elevated cytotoxic T cell response [211]. Although effector T cells in the tumor microenvironment can be quickly exhausted and are unable to easily recover, provoked immune therapies such as adoptive T-cell therapy have been proposed to reinforce tumor-specific T cells [212]. Adoptive T-cell therapy was engineered by identifying and expanding tumor-specific antigen T cells or creating T cells with tumor-specific chimeric antigen receptor (CAR). To elevate the execution of the antitumor effect in adoptive T-cell therapies, increased mitochondrial metabolism by glycolytic or glutamine metabolism inhibition might be beneficial [213, 214]. In addition, recent evidence has demonstrated that CAR-T-cell engineering with PRODH2, a proline dehydrogenase, might improve the mitochondrial function of CD8 + T cells and elevate the antitumor effect in an animal model [215]. Moreover, a mitochondria-targeted small molecule IR-780 was found to induce immunogenic cell death, which might improve the anticancer effect of adoptive T-cell therapy [216]. These results suggest that the CAR-T-cell engineering technique with mitochondrial modulation may be a promising therapeutic modality for cancer immunotherapy.

## Conclusion

**Fig. 1:**
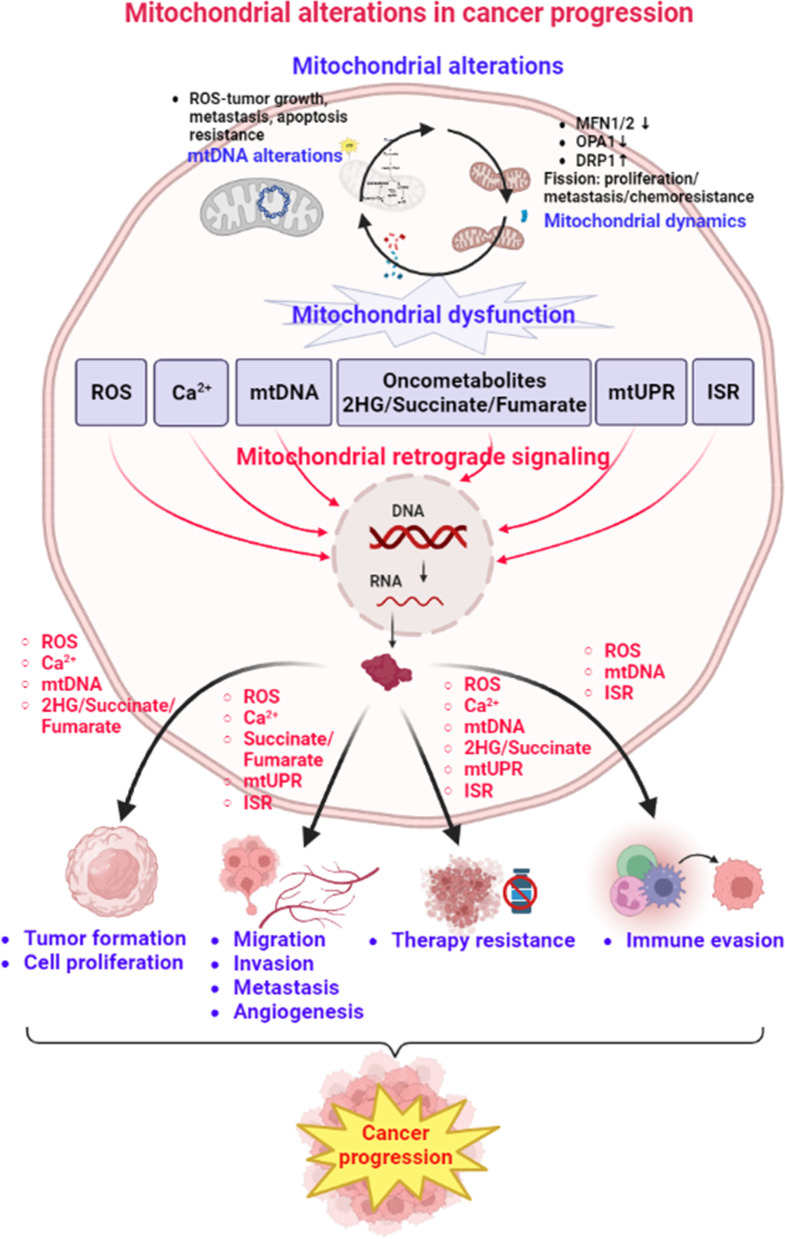
Mitochondrial alterations and mitochondrial retrograde signaling in cancer progression. Several mitochondrial alterations have been implicated in various types of human cancers. Mitochondrial alteration-induced mitochondrial dysfunction might activate mitochondrial retrograde signaling pathways by mitochondrion-derived molecules (ROS, calcium, oncometabolites, and mtDNA) and mitochondrial stress response pathways (mtUPR and ISR) to promote cancer progression to malignancy. The figure was created with BioRender.com

**Fig. 2:**
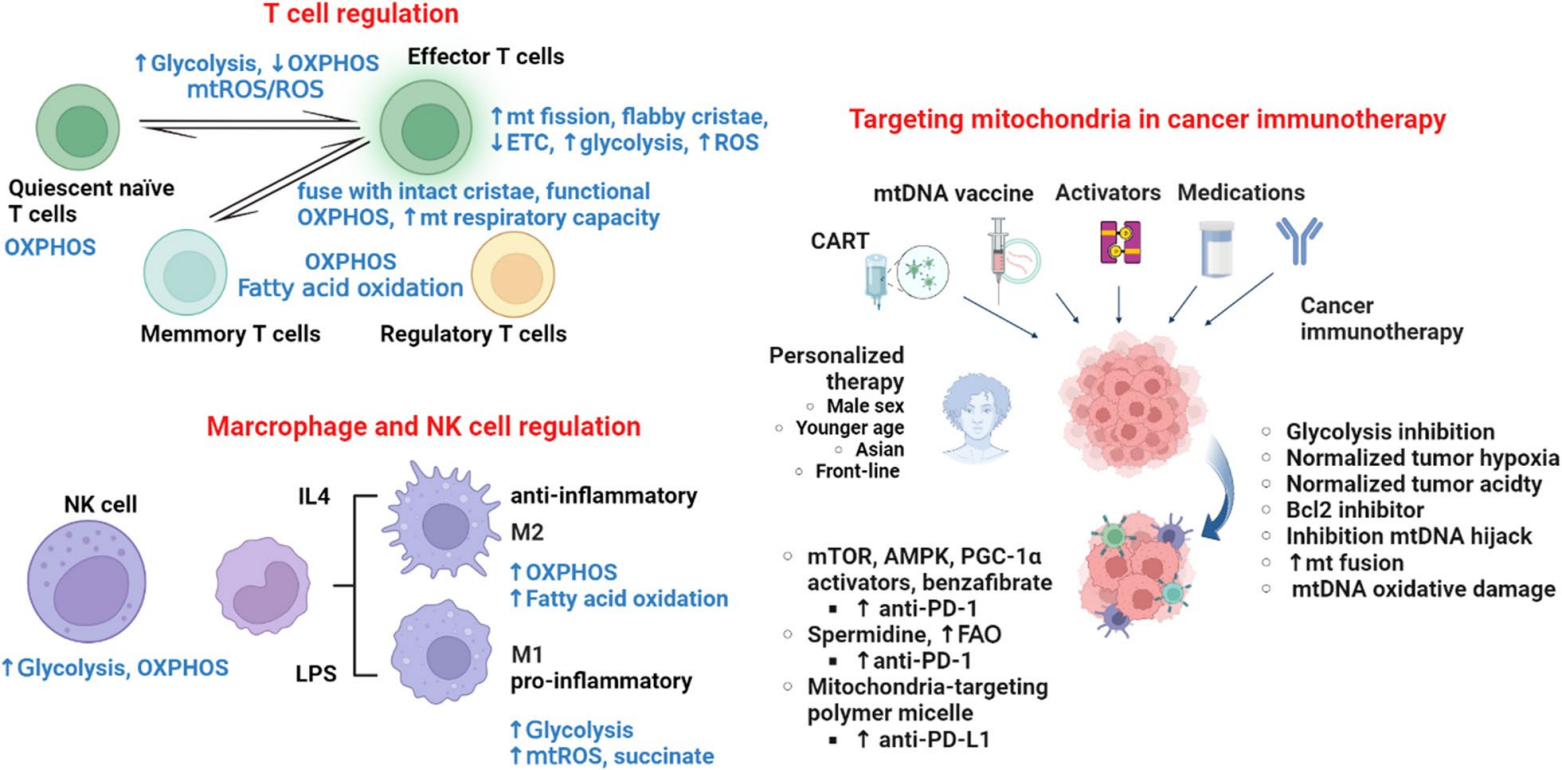
The role of mitochondria in cancer immunity. Mitochondria are essential for the immune regulatory function of T cells, macrophages, and NK cells. In T cells, activated T cells might rely more on glycolysis than OXPHOS and are characterized by fission and flabby cristae-type mitochondria. ROS are essential to the activation of T cells. Switching to OXPHOS or fatty acid oxidation might be implicated in the immunosuppressive status. Similarly, proinflammatory macrophages are more dependent on glycolysis and ROS, while increased OXPHOS and fatty acid oxidation might contribute to the differentiation of anti-inflammatory macrophages. NK cells mainly utilize glucose through elevated glycolysis and OXPHOS to support cytokine secretion and maintain cytotoxic activity. A combination of immune checkpoint inhibitors with agents modulating energy metabolism and mitochondria might be a precision and personalized modality for cancer immunotherapy. The figure was created with BioRender.com

Mitochondria are critical cellular organelles and are responsible for many physiological processes, including cellular metabolism, ROS production, and cell death. Mitochondrial dysfunction has been suggested to contribute to various human diseases, including neurodegenerative diseases and cancer. In the past, several mitochondrial alterations have been identified in cancers, which explain the mechanism underlying the Warburg effect and metabolic reprogramming, as well as promote cancer progression to malignancy. Mitochondrial dysfunction might activate several mitochondrial retrograde signaling pathways by mitochondrion-derived molecules (ROS, calcium, oncometabolites, and exported mtDNA) and mitochondrial stress response pathways (mtUPR and ISR) to promote cancer progression to malignancy (Fig. 1). In addition, mitochondrial functions are essential for the immune regulatory function of immune cells. Changes in energy metabolism or mitochondria might suppress the anticancer functions of immune cells and enhance the immune escape of cancer cells in the tumor microenvironment (Fig. 2). These findings suggest that targeting mitochondrial retrograde signaling in cancer cells and modulating metabolism and mitochondria might be promising therapeutic strategies against cancer progression to malignancy. The cancer-specific differences in mitochondrial alterations, mitochondrial retrograde signaling pathways, and the response to immunotherapy need further investigation to develop precise and personalized medicine against cancer.

## Data Availability

Not applicable.

## Abbreviations

2HG: 2-Hydroxyglutarate
ACC: Acetyl carboxylase
AMPK: AMP-activated protein kinase
ATF4: Activating transcription factor 4
ATP: Adenosine triphosphate
CAR: Chimeric antigen receptor
CTLA-4: Cytotoxic T-lymphocyte-associated protein 4
CPT: Carnitine palmitoyltransferase
DRP1: Dynamin-related protein 1
eIF2α: Eukaryotic translation initiation factor 2α
EMT: Epithelial-to-mesenchymal transition
ETC: Electron transport chain
FH: Fumarate hydratase
GCN2: General control nonderepressible 2
GDF15: Growth differentiation factor 15
GDH: Glutamate dehydrogenase
GLS: Glutaminase
GOT2: Aspartate aminotransferase 2
GPT2: Mitochondrial glutamate-pyruvate transaminase 2
HCC: Hepatocellular carcinoma
HIF-1: Hypoxia inducible factor-1
HRI: Heme-regulated inhibitor kinase
IDH: Isocitrate dehydrogenase
IL: Interleukin
ISR: Integrated stress response
KRAS: Kirsten rat sarcoma viral oncogene homolog
MCU: Mitochondrial calcium uniporter
MFN: Mitofusin
MHC: Major histocompatibility complex
mtDNA: Mitochondrial DNA
mTOR: Mammalian target of rapamycin
mtUPR: Mitochondrial unfolded protein response
NAC: N-acetylcysteine
NK: Natural killer
OPA1: Optic atrophy 1
ORFs: Open-reading frames
OXPHOS: Oxidative phosphorylation
PD-1: Programmed death-1
PD-L1: Programmed cell death 1 ligand 1
PERK: Protein kinase R-like endoplasmic reticulum kinase
PGC-1α: Peroxisome proliferator-activated receptor-gamma coactivator-1α
PHD: Prolyl-hydroxylase domain
PI3K: Phosphatidylinositol 3-phosphate kinase
PKR: Protein kinase R
ROS: Reactive oxygen species
SDH: Succinate dehydrogenase
STAT3: Signal transducer and activator of transcription 3
STING: Stimulator of interferon genes
TCA: Tricarboxylic acid
TET: Ten-eleven translocation
TILs: Tumor-infiltrating lymphocytes
TNBC: Triple-negative breast cancer
α-KG: α-Ketoglutarate
